# Budget impact analysis of neoadjuvant nivolumab for non-small cell lung cancer in the Chilean public healthcare system: An exploratory economic assessment

**DOI:** 10.1007/s12094-025-03872-7

**Published:** 2025-03-02

**Authors:** Daniela Paredes-Fernández, Rony Lenz-Alcayaga, Francisco Orlandi-Jorquera

**Affiliations:** 1 Public Health Institute, Universidad Andrés Bello, Santiago, Chile; 2 National Thorax Institute, Santiago, Chile

**Keywords:** Non-small-cell lung carcinoma, Neoadjuvant therapies, Economics, High-cost technology

## Abstract

**Purpose:**

Effective and sustainable treatments to improve patient outcomes are urgently needed for non-small cell lung carcinoma (NSCLC). Neoadjuvant therapies, particularly nivolumab, have shown superior outcomes in event-free survival and pathological response, yet financial coverage is scarce. We aim to provide an exploratory economic analysis to assess the implications of its incorporation into routine clinical practice.

**Methods:**

We conducted a six-step BIA (budget impact analysis) based on a decision tree model for pathways, probabilities, and resource utilization from the national payer perspective at an event-free survival (EFS) horizon. We estimated the direct cost of drugs and all healthcare-related services for two scenarios: a baseline scenario [neoadjuvant chemotherapy (CT)] and an alternative scenario [neoadjuvant nivolumab combined with chemotherapy (N + CT)].

**Results:**

The funnel-down technique determined 359 eligible patients nationwide per year. The total cost of treatment in the baseline scenario amounts to CLP $ 7315 million Chilean pesos (€ 8,063,219) per cohort, with three top cost drivers: 1L drugs after recurrence (51.98%), resection (29.33%) and 2L nivolumab (5.85%). The alternative scenario amounted to CLP $ 6853 million (€ 7,553,572), with the highest relative expenditure attributed to the N + CT scheme (61.76%), resection (31.31%), and follow-up (2.73%). Adjuvant costs decrease to 1.03%, as does the expenditure on 1L (51.98% versus 0.34%) and 2L treatments (5.85% versus 0.18%). Early intervention in NSCLC reduces the budgetary impact by 6.3% (savings of − $ 462 million (€ 509,647) per treated cohort).

**Conclusions:**

Early incorporation of N + CT optimizes healthcare expenditure by providing access to therapies that improve survival rates while reducing the need for costly treatments in advanced stages. This approach represents a dominant strategy.

## Introduction

Lung cancer remains a significant global healthcare challenge, being the most frequently diagnosed cancer, with nearly 2.5 million new cases. This leading cause of cancer-related mortality is responsible for 18.7% of cancer fatalities [1].

In Chile, more than 27,000 patients have died due to lung cancer in an 8-year observation period [2], and nearly 3500 deaths are recorded annually [3]. Lung cancer is a heterogeneous disease [4]; however, cases are usually diagnosed at advanced stages, and only 10–25% qualify for curative surgical interventions [3].

In 2020, the government incorporated lung cancer care into the national explicit coverage regimen (GES—*Garantías Explícitas en Salud* in Spanish). The GES regimen guarantees treatment options, including surgery, radiotherapy, chemotherapy, and palliative care. The GES regimen covers chemotherapy in various non-small cell lung carcinoma (NSCLC) stages, including IB, IIA, IIB, and IIIA [5]. Experience from a center in southern Chile has shown that although diagnoses were often made at advanced stages before GES, there has been a significant increase in early detection and the frequency of curative surgeries [3]. According to national statistics, the detection of lung cancer within GES increased by 61.43% from 2020 to 2023 in the public sector. Beyond GES coverage, additional funding mechanisms have been established to enhance access to targeted therapies and anti-PD-L1 antibody immunotherapy for second-line treatment of advanced NSCLC.

Given the high burden of disease and increasing early-stage detection, effective and sustainable treatment strategies to improve patient outcomes are urgently needed. Neoadjuvant therapies, particularly nivolumab, an immune checkpoint inhibitor targeting the PD-1 pathway, have shown promise in managing NSCLC before surgical resection. Recently, nivolumab has gained FDA approval for a second indication as a neoadjuvant treatment, in combination with platinum-based chemotherapy, for resectable NSCLC with tumors ≥ 4 cm or positive lymph nodes [6]. 

Forde et al. (2022) explored the potential advantages of incorporating nivolumab as a neoadjuvant alternative. The CheckMate 816 trial, a phase 3 open-label study, randomized patients with resectable stage IB to IIIA NSCLC to receive either nivolumab in combination with platinum-based chemotherapy or platinum-based chemotherapy alone, followed by surgical resection within 6 weeks. The results indicated a median event-free survival (EFS) of 31.6 months (95% CI, 30.2 to not reached) for the nivolumab plus chemotherapy (N + CT) group compared to 20.8 months (95% CI, 14.0–26.7) for the chemotherapy-alone (CT) group. Additionally, the pathological complete response was significantly higher in the N + CT group at 24.0% (95% CI, 18.0–31.0) versus 2.2% (95% CI, 0.6–5.6) in the CT group (odds ratio, 13.94; 99% CI, 3.49–55.75; *P* < 0.001) [7]. In a recent 3-year update of the CheckMate 816 trial, Forde et al. (2023) reported significant improvements in EFS for patients receiving N + CT compared to those receiving CT. At 1 year, the EFS was 77% in the N + CT arm versus 64% in the CT group. By 24 months, the EFS rates were 65% and 47%, respectively, and at 36 months, the EFS was 57% for the N + CT group compared to 43% for the control. The recurrence rate in the N + CT arm was recorded at 28%, significantly lower than the 42% outcome observed in the CT arm. Locoregional recurrences occurred in 19% of patients treated with N + CT, compared to 22% in those receiving CT alone. Furthermore, distant recurrence rates were lower in the N + CT cohort, with 10% experiencing recurrence versus 22% in the CT group. These findings underscore the potential of nivolumab to enhance treatment outcomes in resectable NSCLC [8].

Provencio et al. (2023) conducted a new exploratory subgroup analysis at 36 months, focusing on the expression of the PD-L1 marker. In the subgroup with PD-L1 expression ≥ 1%, the pathological complete response (pCR) was higher, with 32.6% in the N + CT arm compared to 2.2% in CT group. Additionally, EFS showed a hazard ratio of 0.46 (0.28–0.77), with an EFS rate at 12 months of 83% for the combination therapy compared to 62% for the control group. Furthermore, overall survival at 36 months remained significantly higher in the N + CT arm at 85%, compared to 66% in the CT arm [9].

The objective of this study is to provide an exploratory economic analysis through a budget impact analysis of the incorporation of neoadjuvant N + CT for early NSCLC from the perspective of the services provided by the Chilean public healthcare system, aiming to assess the financial implications and potential economic benefits of integrating this immunotherapy into routine clinical practice.

## Methods

We performed a six-step BIA using a decision tree model to evaluate pathways, probabilities, and resource utilization from the national payer perspective over an EFS horizon per cohort. The analysis estimated the direct costs of drugs and healthcare-related services for a baseline scenario with neoadjuvant chemotherapy (CT) and an alternative scenario with neoadjuvant nivolumab plus chemotherapy (N + CT). The following section outlines the detailed methodology for each stage of the analysis.

The study is designed to compare two scenarios: a current baseline scenario in which patients receive neoadjuvant CT followed by surgery and an expected scenario of access to neoadjuvant N + CT followed by surgery.

**Fig. 1:**
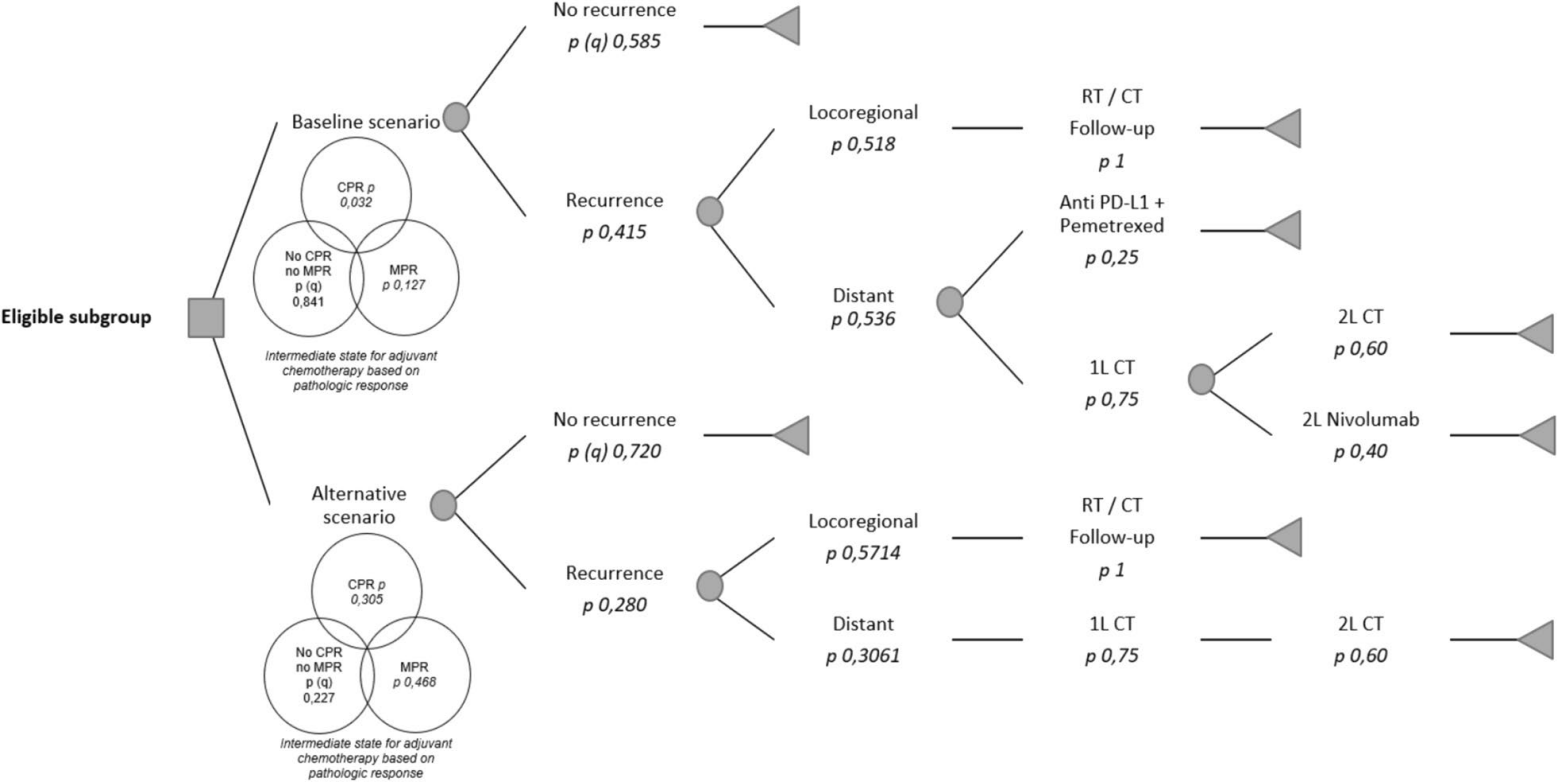
**Decision tree for BIA estimations of incorporating nivolumab into standard neoadjuvant care for NSCLC**. Legend: CPR stands for complete pathologic response (0% residual viable tumor cells in the primary tumor and sample lymph nodes); MPR stands for major pathologic response (≤ 10% residual viable tumor cells in the primary tumor and sample lymph nodes). Parameters used in this model have been retrieved from CheckMate 816 data and National Thorax Institute interim registries. (*q*) stands for the mathematical complement

The analysis organizes these elements into a decision tree model that illustrates the pathways patients may follow based on currently available therapies in Chile and the associated probabilities of each scenario based on the explored evidence. This decision tree enables a detailed evaluation of the potential economic implications of incorporating nivolumab into standard neoadjuvant care for NSCLC as it pragmatically represents associated healthcare resource utilization (Fig. 1).

In the baseline current scenario, eligible patients undergo neoadjuvant CT and surgery. Depending on their pathological response, some patients may require adjuvant CT. Afterward, patients enter a follow-up period during which recurrence may occur. Regardless of recurrence, the national payer must finance follow-up care for up to five years. For patients with locoregional metastasis, radiotherapy or radiochemotherapy (dual or monoenergetic LINAC) is provided, followed by continued monitoring. In distant metastasis cases, treatment decisions are guided by the expression of the PD-L1 marker. Patients with PD-L1 expression above 50%, which has been locally estimated to occur in 25% of cases [10], may receive anti-PD-L1 immunotherapy, with treatment regimens differing based on squamous or non-squamous histology. Based on local coverage, when used as first-line treatment, an assumption of a maximum of 35 cycles with an average weight of 75 kg at a dosage of 75 mg/m^2^ was applied. A differentiated approach was adopted for patients with squamous versus non-squamous histology. Four cycles of pemetrexed and cisplatin are considered, as well as maintenance pemetrexed for 13 months (17.3 cycles) in the case of non-squamous histology. For squamous histology, a regimen of carboplatin and paclitaxel for four cycles is recommended. For patients with PD-L1 expression below 50%, first-line chemotherapy (1L CT) is administered according to histological subtype. Second-line chemotherapy involves a single-agent drug for four cycles, with the option of nivolumab as a second-line therapy at standard dosing with a standard treatment of 240 mg every 2 weeks or 360 mg every 3 weeks until disease progression for patients with > 1% of PD-L1 expression.

In the alternative scenario, patients would have access to neoadjuvant N + CT, and they may still require adjuvant CT, though at lower rates, due to improved complete and major pathological responses. The recommended dosage of nivolumab for this indication was used: 360 mg every 21 days for three cycles. For those patients who remain under observation, recurrence or non-recurrence is assessed, with the latter group continuing in routine periodic follow-up. In cases of recurrence, patients may experience locoregional recurrence and subsequently receive radiotherapy or radiochemotherapy regimens. For those with distant metastasis, 1L CT will be administered based on histology, while a subset of patients may receive second-line single-agent CT.

The present study employs the six-step budget impact analysis (BIA) approach outlined by the International Society for Pharmacoeconomics and Outcomes Research (ISPOR). The steps comprise estimating the target population, selecting a time horizon, identifying the current and projected treatment mix, estimating current and future drug costs, estimating changes in disease-related costs, and estimating and presenting changes in annual budget impact and health outcomes [11].

### Estimating the target population

**Fig. 2:**
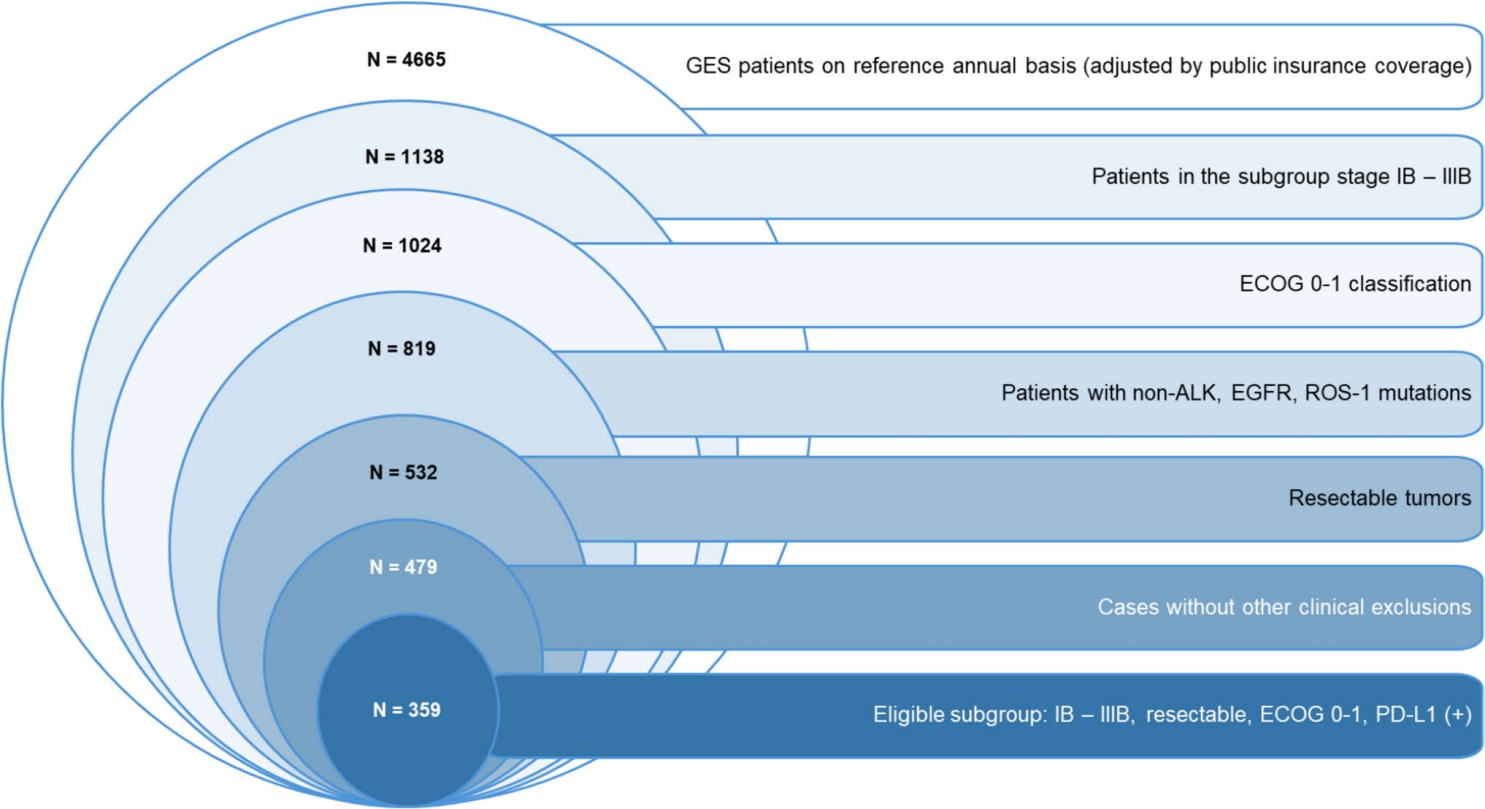
**Funnel-down technique for estimating target population based on eligibility criteria per cohort.** Legend: Patients under GES lung cancer program were identified using publicly available national databases, including all newly diagnosed cases over 12 months (Source: National Superintendency. GES July 2005–September 2024) Trimestral Statistics) [14]. The data was further adjusted by disease stage, using distributions based on patients from the National Thorax Institute, which serves as the national lung cancer reference center. Cases were distributed as follows: Stage I (16.0%), IA (12.8%), IB (3.2%), low-risk IB (1.6%), high-risk IB (1.6%), Stage II (10.0%), IIIA (2.0%), IIIB (10.8%), and IIIC–IV (61.2%). These stage distributions were combined with ECOG scores, considering 90.0% of cases with a score of 0–1. Mutations in ALK (5.0%), EGFR (15.0%), and ROS-1 (1.0%) were excluded from the target population. For the remaining subgroup, 65.0% were eligible for resection, and an additional 10.0% adjustment was applied to account for exclusions due to other clinical factors and voluntary treatment discontinuation (Source: National Thorax Institute Oncology Committee interim data)

The eligible population for this analysis was identified using a funnel-down technique based on Earnshaw and Mauskof (2017) recommendations [12]. This begins with the national population and involves several adjustments. First, the analysis adjusts for the differing national incidence rates of NSCLC between men and women [13] and accounts for the annual cases reported in the public healthcare sector [14]. The methodology incorporates adjustments based on disease stages, leveraging data from the National Thorax Institute to distribute patients according to their clinical attributes. Additional adjustments include the Eastern Cooperative Oncology Group (ECOG) performance status and exclusionary mutations limiting the assessed combination. Finally, the analysis was adjusted by the PD-L1 expression levels and the characteristics of resectable tumors based on interim data from the National Thorax Institute. This approach ensures that the selected population of 359 subjects reflects a realistic and clinically relevant cohort for assessing the economic implications of neoadjuvant nivolumab in NSCLC in the public sector in Chile (Fig. 2).

### Selecting a time horizon

The time horizon selected for this analysis considers within trial data, without beyond-trial extrapolations, to control parametric uncertainty. Thus, the horizon for capturing costs and economic impact is 36 months per cohort. Based on Earnshaw and Mauskof (2017) recommendations, the model has been calibrated by EFS. Given the target population and reported survival rates for NSCLC, an incidence and cohort modeling approach was deemed appropriate.

### Identifying the current and projected treatment mix

Current therapeutic options for this subgroup include resection, neoadjuvant CT, radiotherapy, chemoradiotherapy, histology-based CT, immunotherapy in first and second line, and palliative care. In contrast, the projected treatment mix would encompass neoadjuvant N + CT, surgical resection, radiotherapy, chemoradiotherapy, histology-based CT, and palliative care.

### Estimating the current and future drug costs

To estimate drug-related costs, posology and event-free survival (EFS) data were necessary to adjust actual consumption during the modeled period. The usage rates and treatment cycles were based on those outlined in the model. Pricing information was gathered from multiple sources, reflecting that expenditures and purchasing for these technologies are funded through various financial sources. Resection costs were retrieved from the National Thorax Institute Diagnosis-Related Groups (DRG) database. Using a Venn algorithm, data was sorted by ICD-9-MC codes related to the intervention and associated with a CIE-10 lung cancer diagnosis. The average annual exchange rate observed in 2023, according to the Central Bank of Chile, was used. We present the following metadata chart to report costs applied in the model. We also combine specific clinical data retrieved to support the model's parameters and transitions (Table 1).

**Table 1:** Cost and clinical metadata

Cost metadata		
Cost driver	Parameter CLP (€)	Comment
Resection	$ 5,970,820 (€ 6582)	Source: DRG National Thorax Institute. Data based on *n* = 286 discharges for ICD-9-MC codes of minimally invasive surgery and thoracotomy: 32.41; 32.49; 32.30; 32.39 associated with lung cancer diagnosis codes. Request No. AO105T0000331
Neoadjuvant CT	$ 865,309 (€ 954)	Adapted from Ministry of Health. 2022. Verification Study of the Expected Average Individual Cost per Beneficiary for the Prioritized Set of Health Problems with Explicit Guarantees. Cost for complete treatment
Adjuvant CT	$ 865,309 (€ 954)	
1L Checkpoint Inhibitor	$ 5,300,540 (€ 5,843)	Adapted from Ministry of Health. 2022. Verification Study of the Expected Average Individual Cost per Beneficiary for the Prioritized Set of Health Problems with Explicit Guarantees. Cost per cycle
Pemetrexed	$ 149,730 (€ 165)	
Carboplatin	$ 149,730 (€ 165)	
Docetaxel	$ 123,150 (€ 136)	
Standard radiotherapy with monoenergetic Linac	$ 2,476,640 (€ 2730)	Adapted from Ministry of Health. 2022. Verification Study of the Expected Average Individual Cost per Beneficiary for the Prioritized Set of Health Problems with Explicit Guarantees. Schemes are equally utilized in cases with locoregional recurrence
Complex radiotherapy with dual Linac	$ 2,980,780 (€ 3286)	
2L nivolumab in metastatic indication	$ 17,121,868 (€ 18,873)	Estimation based on recommended standard treatment: 240 mg every 15 days, according to PFS reported by Source: Calpe-Armero, P. et al. 2017. Effectiveness of nivolumab versus docetaxel as second-line treatment in non-small cell lung cancer patients in clinical practice. *Chemotherapy*. 2017;62(6):374–380
Nivolumab in neoadjuvant scheme	$ 10,913,361 (€ 12,030)	Estimation based on recommended standard treatment: 360 mg for three doses every 21 days in CheckMate 816
Histology-based CT for squamous non-small cell lung cancer	$ 682,230 (€ 752)	Adapted from Ministry of Health. 2022.Verification Study of the Expected Average Individual Cost per Beneficiary for the Prioritized Set of Health Problems with Explicit Guarantees. Cost for complete treatment Considers full drug schemes combination
Histology-based CT for Non-squamous non-small cell lung cancer	$ 535,210 (€ 590)	
First-year follow-up	$ 705,190 (€ 777)	Adapted from Ministry of Health. 2022. Verification Study of the Expected Average Individual Cost per Beneficiary for the Prioritized Set of Health Problems with Explicit Guarantees. Annual cost of CT-scan and oncologist appointments
Second-year follow-up	$ 194,000 (€ 214)	
Third- to 5th-year follow-up	$ 97,000 (€ 107)	

Clinical metadata		
Clinical parameter	Parameter	Comment
Recurrence after surgery with CT	41.5%	Based on CheckMate 816 trial; median follow-up of 41.4 months. Source: Forde PM, et al. 84O Neoadjuvant nivolumab (N) + platinum-doublet chemotherapy (C) for resectable NSCLC: 3-y update from CheckMate 816. Journal of Thoracic Oncology. 2023;18: S89–S90
No recurrence after surgery with CT	58.5%	
Locoregional recurrence after CT	51.8%	Based on CheckMate 816 trial. Source: Supplement to Forde PM, et al. Neoadjuvant Nivolumab plus Chemotherapy in Resectable Lung Cancer. *New England Journal of Medicine*. 2022;386: 1973–1985. 10.1056/NEJMoa2202170
Distant recurrence after CT	53.6%	
Recurrence with N + CT	28.0%	
No recurrence after surgery with N + CT	72.0%	
Locoregional recurrence with N + CT	57.1%	
Distant recurrence with N + CT	30.6%	
Complete pathological response to CT	3.2%	
Major pathological response to CT	12.7%	
Complete pathological response to N + CT	30.5%	
Major pathological response to N + CT	46.8%	
Patients without CPR or MPR after CT	84.1%	Methodological assumption to estimate adjuvant CT needs
Patients without CPR or MPR after N + CT	22.7%	
Event-free survival N + CT12 mo	77.0%	Based on CheckMate 816 trial Median. Source: Forde PM, et al. 84O Neoadjuvant nivolumab (N) + platinum-doublet chemotherapy (C) for resectable NSCLC: 3-y update from CheckMate 816. Journal of Thoracic Oncology. 2023;18: S89–S90
Event-free survival CT y 12 mo	64.0%	
Event-free survival N + CT 24 mo	65.0%	
Event-free survival CT 24 mo	47.0%	
Event-free survival N + CT 36 mo	57.0%	
Event-free survival CT 36 mo	43.0%	
Squamous cell histology proportion	20.0%	Source: National Thorax Institute Oncology Committee interim data
Non-squamous cell proportion	80.0%	

This economic analysis was performed without relying on individual patient data for the decision tree model, and only secondary data was used.

## Results

Based on the six-step approach, we present the change in disease-related costs and budget impact for a cohort sample.

In the current baseline scenario (neoadjuvant CT and the rest of the cost drivers), the total cost of NSCLC treatment amounts to $ 7315 million (€ 8,063,219) for 359 eligible patients per cohort. This encompasses various cost drivers, including neoadjuvant CT, resections, adjuvant therapy, follow-up, and costs associated with recurrence treated with 1L and 2L therapies. In the alternative scenario, the total cost of NSCLC care incorporating neoadjuvant N + CT amounts to $ 6853 million (€ 7,553,572). This scenario includes costs of neoadjuvant CT, resection, adjuvant CT, follow-up, and recurrence treatment such as radiotherapy, 1L, and 2L CT.

Early intervention in NSCLC allows for correcting spending trajectories and financial architecture by reducing the budgetary impact by 6.3%, equivalent to savings of − $ 462 million (€ 509,647) per treated cohort (Table 2).

**Table 2:** Cost estimations of baseline scenario: neoadjuvant CT and cost drivers versus alternative scenario: neoadjuvant N + CT

Baseline scenario			
Cost driver	Patients per costs driver (patient basis)	Average cost per patient in the cohort	Total cost per driver
Neoadjuvant CT	359	$ 865,309 (€ 954)	$ 310,921,003 (€ 342,721)
Resection	359	$ 5,970,820 (€ 6,582)	$ 2,145,422,345 (€ 2,364,851)
Adjuvant CT	302	$ 865,309 (€ 954)	$ 261,568,463 (€ 288,321)
Follow-up of non-recurrent patients	210	$ 584,212 (€ 644)	$ 122,840,716 (€ 135,405)
Locoregional recurrence	77	$ 3,161,365 (€ 3485)	$ 244,015,627 (€ 268,973)
Distant metastasis treatment, 1L checkpoint Inhibitor	20	$ 188,789,003 (€ 208,098)	$ 3,768,625,889 (€ 4,154,072)
Distant metastasis treatment, 1L CT	60	$ 564,614 (€ 622)	$ 33,812,652 (€ 37,271)
Distant metastasis treatment, 2L CT	36	$ 492,600 (€ 543)	$ 17,699,999 (€ 19,510)
Distant metastasis treatment, 2L checkpoint Inhibitor	24	$ 17,121,868 (€ 18,873)	$ 410,146,239 (€ 452,095)
Baseline scenario costs	$ 7,315,052,933 (€ 8,063,219)		

Alternative scenario			
Cost driver	Patients per costs driver (patient basis)	Average cost per patient in the cohort	Total cost per driver
Neoadjuvant CT	359	$ 865,309 (€ 954)	$ 310,921,003 (€ 342,721)
Neoadjuvant N	359	$ 10,913,361 (€ 12,030)	$ 3,921,366,004 (€ 4,322,434)
Resection	359	$ 5,970,820 (€ 6582)	$ 2,145,422,345 (€ 2,364,851)
Adjuvant CT	82	$ 865,309 (€ 954)	$ 70,563,632 (€ 77,781)
Follow-up of non-recurrent patients	259	$ 724,386 (€ 798)	$ 187,405,168 (€ 206,573)
Locoregional recurrence	57	$ 3,161,365 (€ 3485)	$ 181,749,571 (€ 200,338)
Distant metastasis treatment, 1L CT	41	$ 564,614 (€ 622)	$ 23,149,174 (€ 25,517)
Distant metastasis treatment, 2L CT	25	$ 492,600 (€ 543)	$ 12,117,960 (€ 13,357)
Alternative scenario costs	$ 6,852,694,856 (€ 7,553,572)		
Differential expenditure between scenarios	$ − 462,358,077 (€ 509,647) = − 6.3%		

**Fig. 3:**
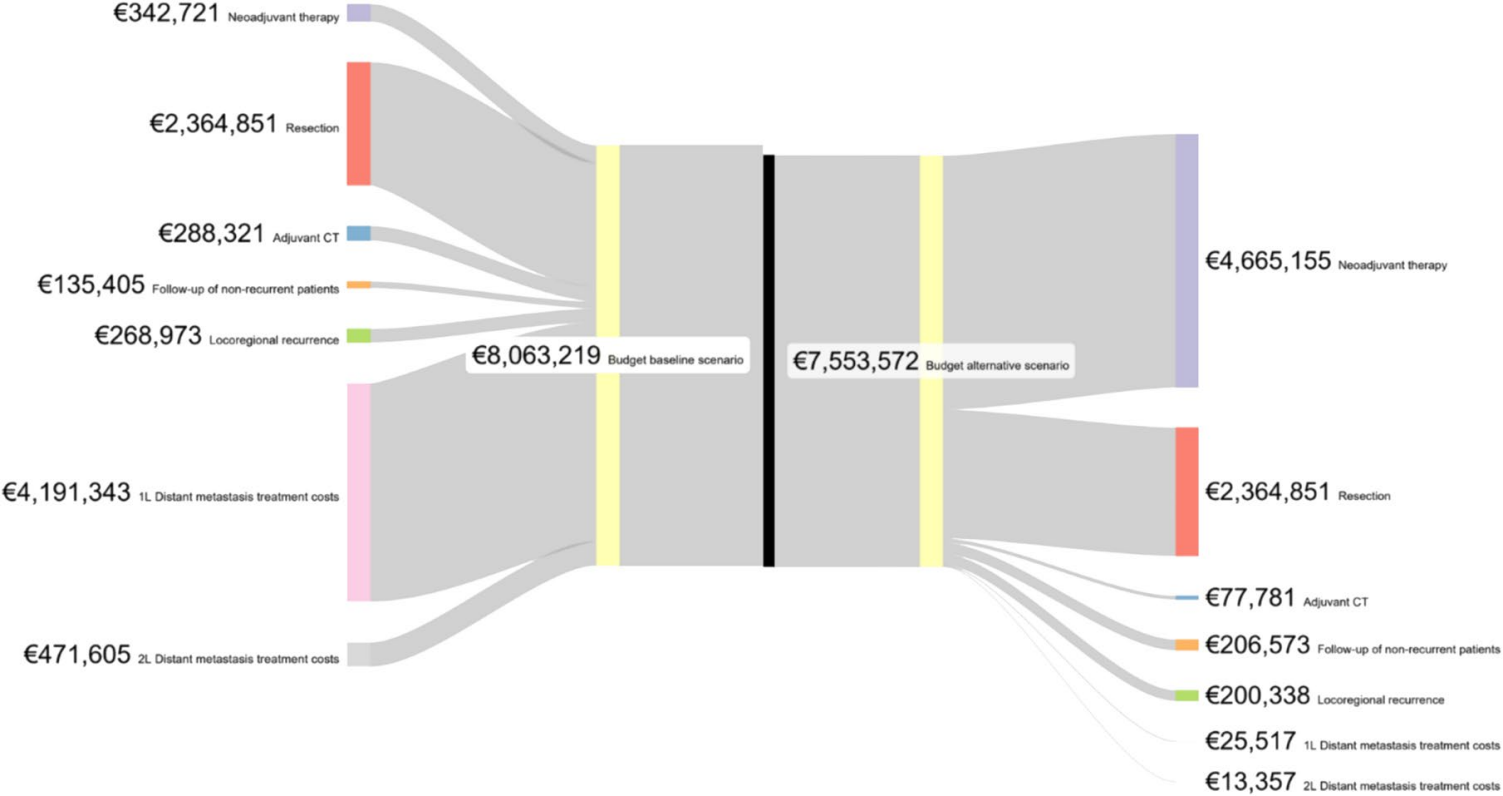
Sankey diagram comparing cost driver distribution of baseline scenario (neoadjuvant CT) versus alternative scenario (neoadjuvant N + CT). For representation, costs have been grouped by type and line of treatment

For better appreciation, we present a Sankey diagram of each scenario and the cost (in euros) distribution in Fig. 3.

**Fig. 4:**
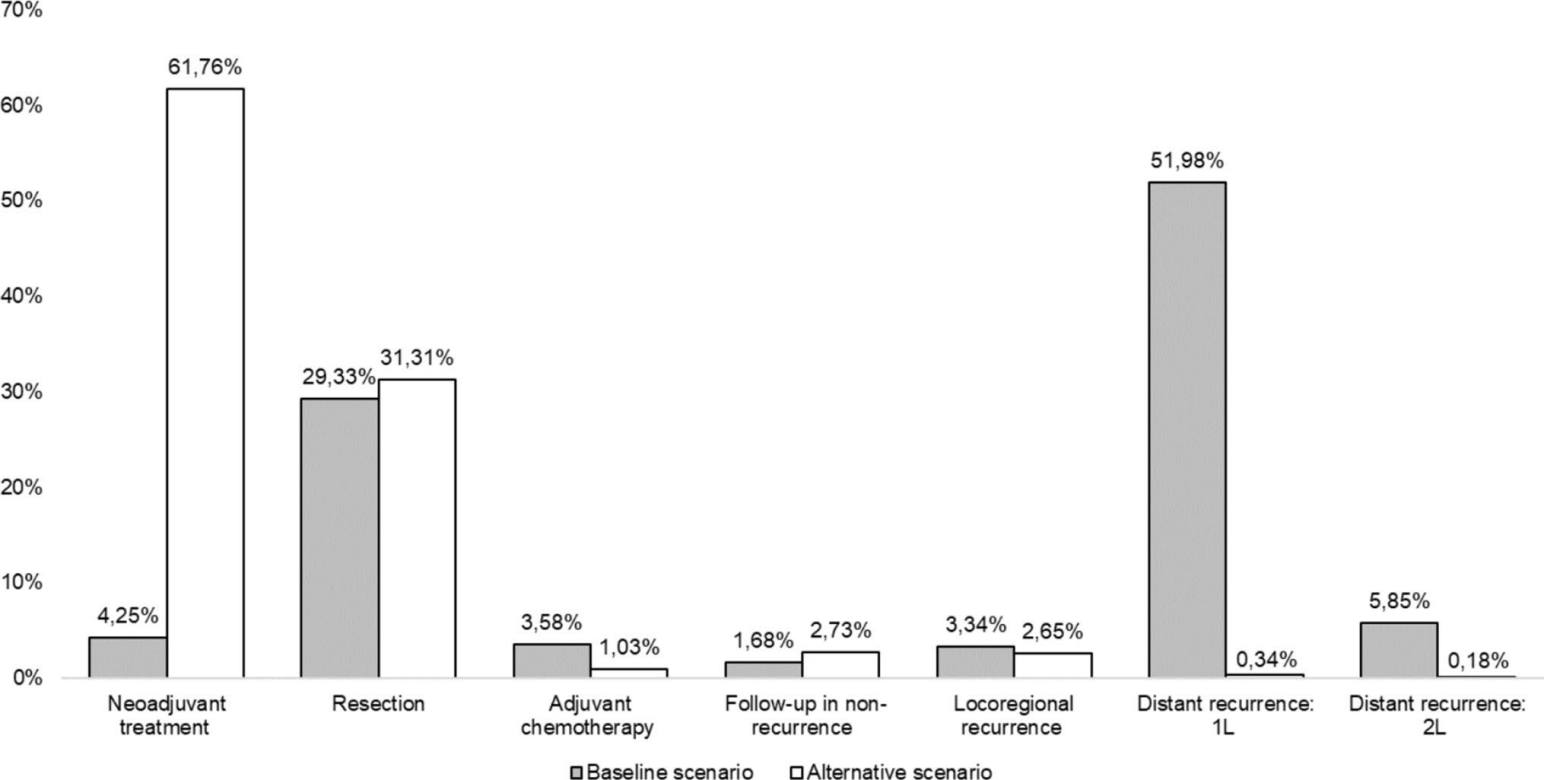
Changes in expenditure participation of each cost driver in each scenario (relative weight of total costs per scenario)

In the baseline scenario, the highest relative weight of total expenditure per cohort is attributed to first-line therapies after recurrence (histology-based CT and 1L checkpoint inhibitors), accounting for 51.98%. Resection represents 29.33%, while the expenditure on second-line treatments with monotherapy and nivolumab constitutes 5.85%. Introducing neoadjuvant N + CT in the alternative scenario corrects the expenditure trajectory, since it is allocated in early stages with greater survival and non-recurrence benefits. The highest relative expenditure is attributed to the N + CT scheme, which accounts for 61.76%. Conversely, the relative expenditure on adjuvant drugs decreases to 1.03%, as does the relative expenditure on 1L (51.98% versus 0.34%) and 2L treatments (5.85% versus 0.18%), reflecting the improved event-free survival profile associated with this indication and decrease in late interventions (Fig. 4).

**Fig. 5:**
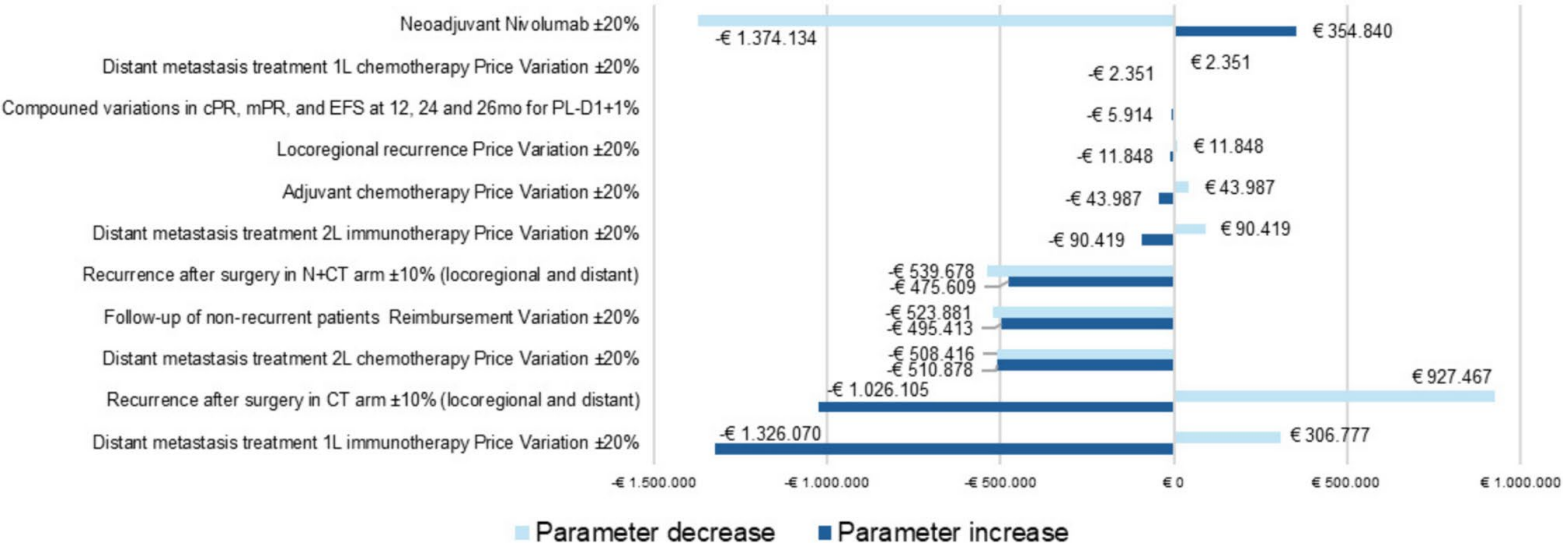
Tornado diagram for deterministic sensitivity analysis: incremental difference between scenarios (in euros)

A deterministic sensitivity analysis was conducted. The model was highly sensitive to the effect of changes in the price of neoadjuvant nivolumab, distant metastasis treatment based on 1L immunotherapy in the baseline scenario, and recurrence rates in the baseline scenario. We also tested the effect of variation of parameters whose improvements were reported in the exploratory subgroup analysis with PD-L1 expression ≥ 1%. For instance, complete pathological response rates with chemotherapy were noted to be 2.2%, while those with nivolumab plus chemotherapy were reported to be 32.6%. Furthermore, EFS rates at 12 months were 62% versus 83%, at 24 months 52% versus 76%, and at 36 months 47% versus 72%, with a hazard ratio of 0.46 (0.28–0.77) based on the 3-year update of the CheckMate 816 study (Provencio et al. 2023). With these adjustments, the variation from the base case amounts to − 6.4%. Parameters such as price variations of neoadjuvant CT or surgery reimbursement rates were not tested, since variations were neutral for incremental purposes (Fig. 5).

## Discussion

The National Institute for Health and Care Excellence (NICE) has recently evaluated neoadjuvant N + CT in resectable NSCLC as part of their health technology assessment process. The External Assessment Group (EAG) cost-effectiveness analysis highlighted several uncertainties, including survival modeling beyond the follow-up CheckMate 816 trial period, curative methodological assumptions, utility estimates related to EFS, locoregional recurrence rates, and treatment restrictions. Despite these uncertainties, incorporating N + CT was considered innovative and cost-effective [15].

Exploratory studies examining the economic profile of neoadjuvant therapies in early-stage high-prevalence cancers were reviewed, such as breast cancer. A cost-effectiveness analysis comparing neoadjuvant pembrolizumab with CT in early-stage cancer demonstrated a favorable economic profile in the USA [16] and Switzerland [17]. In Italy, a cost-consequence evaluation of a neoadjuvant high-cost regimen in early-stage breast cancer suggests that, despite higher initial expenditures, the combination could lead to future savings in both direct and indirect costs by reducing recurrence-related expenditures [18].

As a premise, reducing disease recurrence can potentially improve healthcare expenditure in NSCLC [19]. In the USA, evaluation favors neoadjuvant pembrolizumab compared to only offering adjuvant treatments, highlighting the added quality-adjusted life years achieved when treatments are provided early [20]. An exploratory cost-effectiveness study evaluating perioperative immunotherapy in NSCLC concluded that neoadjuvant nivolumab demonstrated superiority over other regimens in patients with PD-L1 expression ≥ 50%, including other immunotherapies and standard of care [21]. A cost-effectiveness study conducted in Japan assessed the incremental cost-effectiveness ratio of perioperative neoadjuvant therapy with nivolumab and atezolizumab, demonstrating a favorable economic profile increasing quality-adjusted life years while at a positive threshold [22].

In Italy, the LIFE study on the economic burden of NSCLC demonstrated higher expenditures as treatment lines progressed with higher participation of target drugs until the late stage of the disease [19]. In Spain, the costs of distant recurrence in NSCLC are fivefold higher than in locoregional recurrence [23]. In the USA, patients who experienced recurrence faced a 6.65 (*P* < 0.001) times higher economic burden, exhibiting increased outpatient visits, hospitalizations, medical consultations, and emergency room visits [24].

As part of the limitations of our work, and due to a pragmatic and relevant incremental costs approach, our analysis does not consider the costs of adverse reactions since CheckMate 816 reports 11% of grade three and four adverse events in the N + CT arm versus 10% in CT. Additionally, the model is highly sensitive to the assumptions and cycles estimated for 1L and 2L treatments. Readers should exercise caution when extrapolating these results to their settings if dosages or discontinuation horizons differ. Finally, thresholds are widely established in cost-effectiveness analysis, but poorly described for BIA. In 2017, NICE defined a £20 million threshold for the first 3 years [25]; nonetheless, each country defines its own thresholds. Based on this limitation, there are no local references for the valuation of the magnitude of these results to determine if these are low, moderate, or high. Another limitation of our analysis is given by the 3-year horizon approach applied to the cohort and no extrapolations were applied to extended horizons. A recent publication with a 4-year follow-up period from CheckMate 816 confirms long-term survival benefits, with a median EFS of 43.8 versus 18.4 months for N + CT vs. CT alone (HR [95% CI], 0.66 [0.49–0.90]) [26]. Future analyses incorporating these gains and additional evidence on long-term recurrences may demonstrate additional economic impacts derived from neoadjuvant N + CT and could expand the estimations beyond the 3-year follow-up horizon used in this exploratory evaluation.

Additionally, Chile lacks a lung cancer registry to capture real-world data on the clinical heterogeneity of these cases. However, to address this limitation, this study incorporates available data from the national reference center for this cancer through the funnel approach, utilizing the best interim statistics on case composition and public payer databases. Finally, since this evaluation is conducted from the national payer perspective, our analysis includes direct costs only, excluding indirect costs and patient-related impacts such as out-of-pocket expenses and quality of life. This limitation may lead to an underestimation of the total socioeconomic impact of the treatment. Future economic evaluations could address this by adopting a broader societal perspective.

## Conclusion

Lung cancer has become a significant public health concern. This targeted approach focuses on patients meeting strict eligibility criteria, ensuring that the resources are efficiently allocated to those who would benefit the most. By optimizing the early timing of immunomodulators in the treatment process, this strategy not only improves clinical outcomes, but also manages healthcare costs more effectively.

The early incorporation of immune checkpoint inhibitors as a neoadjuvant treatment offers a strategy to optimize healthcare expenditures by providing access to therapies that improve survival rates while reducing the need for costly treatments in advanced stages with lower gains in survival. This approach represents a dominant strategy in managing and correcting lung cancer expenditure.
